# Human Papillomavirus Deregulates the Response of a Cellular Network Comprising of Chemotactic and Proinflammatory Genes

**DOI:** 10.1371/journal.pone.0017848

**Published:** 2011-03-14

**Authors:** Rezaul Karim, Craig Meyers, Claude Backendorf, Kristina Ludigs, Rienk Offringa, Gert-Jan B. van Ommen, Cornelis J. M. Melief, Sjoerd H. van der Burg, Judith M. Boer

**Affiliations:** 1 Center for Human and Clinical Genetics, Leiden University Medical Center, Leiden, The Netherlands; 2 Department of Immunohematology and Blood Transfusion, Leiden University Medical Center, Leiden, The Netherlands; 3 Department of Clinical Oncology, Leiden University Medical Center, Leiden, The Netherlands; 4 Department of Microbiology and Immunology, The Pennsylvania State University College of Medicine, Hershey, Pennsylvania, United States of America; 5 Laboratory of Molecular Genetics, Leiden Institute of Chemistry, Gorlaeus Laboratories, Leiden University, Leiden, The Netherlands; 6 Netherlands Bioinformatics Centre, Nijmegen, The Netherlands; University of Hong Kong, Hong Kong

## Abstract

Despite the presence of intracellular pathogen recognition receptors that allow infected cells to attract the immune system, undifferentiated keratinocytes (KCs) are the main targets for latent infection with high-risk human papilloma viruses (hrHPVs). HPV infections are transient but on average last for more than one year suggesting that HPV has developed means to evade host immunity. To understand how HPV persists, we studied the innate immune response of undifferentiated human KCs harboring episomal copies of HPV16 and 18 by genome-wide expression profiling. Our data showed that the expression of the different virus-sensing receptors was not affected by the presence of HPV. Poly(I:C) stimulation of the viral RNA receptors *TLR3*, *PKR*, *MDA5* and *RIG-I*, the latter of which indirectly senses viral DNA through non-self RNA polymerase III transcripts, showed dampening in downstream signalling of these receptors by HPVs. Many of the genes downregulated in HPV-positive KCs involved components of the antigen presenting pathway, the inflammasome, the production of antivirals, pro-inflammatory and chemotactic cytokines, and components downstream of activated pathogen receptors. Notably, gene and/or protein interaction analysis revealed the downregulation of a network of genes that was strongly interconnected by IL-1β, a crucial cytokine to activate adaptive immunity. In summary, our comprehensive expression profiling approach revealed that HPV16 and 18 coordinate a broad deregulation of the keratinocyte's inflammatory response, and contributes to the understanding of virus persistence.

## Introduction

Cervical cancer is the second most common cancer in women worldwide. More than 520,000 women are diagnosed with invasive cervical cancer each year [1]. Cervical and other anogenital carcinomas arise as result of an uncontrolled persistent infection with a high-risk type human papillomavirus (HPV), in particular types HPV16 and HPV18 [2], [3]. A detectable cervicovaginal HPV infection in young women is close to 1–2 years [4] before it is cleared, suggesting that HPV can evade host immunity. Indeed, the infection cycle of HPV is one in which viral replication and release is not associated with overt inflammation [5], [6] and HPV-specific adaptive immune responses are often weak or lacking in patients with progressive HPV infections [7]–[10].

Stratified squamous epithelia consist of undifferentiated (basal layer) and increasingly differentiated KCs. The basal KCs are the primary target of HPV infection [11]. In these cells, innate immunity acts as the first line of defense against invading viruses. KCs express pathogen recognition receptors (PRRs) including TLR9, which responds to viral DNA [12], as well as TLR3, protein kinase R (EIF2AK2), and the RNA helicases RIG-I (DDX58) and MDA5 (IFIH1), which recognize single-stranded and double-stranded RNA (dsRNA) [13]. Ligand binding to these PRRs leads to direct NF-kappa-B activation resulting in the upregulation of pro-inflammatory cytokines, and/or activation of type I interferon (IFN) response genes including transcription factors IRF3 and IRF7 regulating the production of antiviral cytokines [13]–[22].

Expression of specific viral oncoproteins, E6 and E7, is required for maintaining the malignant growth of cervical cancer cells [23]. To understand how HPV infection may alter KCs and evade PRR activation, direct protein interactions including the binding of the HPV E6 oncoprotein to IRF3 have been studied [24], [25]. An OncoChip expression study showed that retrovirally expressed E6 and E7 efficiently downregulated type I IFN responses in keratinocytes, but surprisingly also upregulated the expression of pro-inflammatory cytokines [26]. Another early microarray study described downregulation of interferon-inducible genes in KCs containing episomal HPV type 31 [27]. These studies indicated that HPV-derived proteins could meddle with host immunity but the full spectrum of interference is within the limitations of these studies not visible.

We aimed at understanding the effects of high-risk HPVs on the immune response in KCs. First, we confirmed expression of the viral RNA receptors in undifferentiated and differentiated cells, while DNA sensor *TLR9* was restricted to differentiated cells, and showed that HPV does not interfere with expression levels of the PRRs. Next, we focused our studies on undifferentiated KCs, since these are the target cells for latent infection with HPV. We generated expression profiles of several different control KCs and KCs harboring episomal copies of entire HPV16 or 18 genomes [28], [29] on microarrays representing 24,500 well-annotated transcripts to study differences in the baseline gene expression by the presence of HPV. In addition, we studied differences in response to triggering the viral RNA PRRs with the synthetic dsRNA poly(I:C). Although HPV is a DNA virus, non-self dsDNA can serve as template for transcription into dsRNA by polymerase III and induce type I interferon and NF-Kappa-B through the RIG-I pathway [30]–[32]. Here, we show that HPVs were able to dampen a network of genes associated with activation of the adaptive immune response encoding antimicrobial molecules, chemotactic and pro-inflammatory cytokines, and proteins that are involved in antigen presentation, and that most of them are interconnected via *IL1B*.

## Materials and Methods

### Ethics statement

The use of discarded human foreskin, cervical and vaginal keratinocyte tissues to develop cell lines for these studies was approved by the Institutional Review Board at the Pennsylvania State University College of Medicine and by the Institutional Review Board at Pinnacle Health Hospitals. The Medical Ethical Committee of the Leiden University Medical Center approved the human tissue sections (healthy foreskin, healthy cervix, HPV16- or 18-positive cervical neoplasias) used for staining. All sections and cell lines were derived from discarded tissues and de-identified, therefore no informed consent was necessary.

### Cell culture

Human epidermal KCs were isolated from foreskin, vagina, or cervix of unrelated donors [33] and established on a layer of lethally ^137^Cs-irradiated mouse 3T3 fibroblasts. Passage 4–5 of primary KCs - devoid of contaminating cells - were grown in serum-free medium (Defined KSFM, Invitrogen, Breda, The Netherlands). Partial differentiation was induced by 1.8 mM Ca2+ for 24 hrs, terminal differentiation by placing KCs in single-cell suspension into serum-free medium containing 1.75% methylcellulose and 1.8 mM Ca2+ for 24 hrs [33]. KC cell lines maintaining episomal copies of HPV16 and HPV18 were created via an electroporation technique described previously [28], [29] but without antibiotic selection. The cell lines were 100% HPV-positive. Southern analyses confirmed the recircularization and subsequent maintenance of episomal viral genomes at approximately 50–100 copies per cell (data not shown). The HPV-positive lines growed at similar rates with population doubling times of ∼2 days) and, when placed in raft culture, all underwent the late stages of the virus life cycle, such as genome amplification, late gene expression, and virus production (data not shown). HPV-positive cells were grown in monolayer culture using E medium in the presence of mitomycin C-treated 3T3 fibroblasts [28], [29] for passage 6–7, and adapted to serum-free medium for one passage before experimentation. All cells used were tested and found free of mycoplasm. Where indicated, cells were stimulated with poly(I:C) (25 µg/ml, InvivoGen, San Diego, USA). CCL5 and IL-1B concentrations in supernatants were determined using the Quantikine ELISA kits (R&D Systems, Minneapolis, USA).

### Immunohistochemistry

Standard immunohistochemical staining was performed using antibodies against human RNASE7 (Sigma-Aldrich, Zwijndrecht, Netherlands, dilution 1∶1600) and TLR9 (clone 26C593.2, Imgenex, San Diego, USA, 1∶800). Four-µm sections of formalin-fixed, paraffin-embedded tissues were deparaffinized, endogenous peroxidase was quenched with 0.3% H2O2 in methanol for 20 minutes, and antigen retrieval was performed by boiling the sections for 10 minutes in Tris-EDTA buffer (pH 9.0). For TLR9 antibody stainings, antigen retrieval was performed by boiling the sections for 10 minutes in citrate buffer (pH 6.0). Isotype control antibody against mouse IgG1 (1∶1000 dilution, code X0931, DAKO, Glostrup, Denmark) was used. Primary antibodies were incubated overnight at room temperature. The Powervision detection system was applied (DAKO, Heverlee, Belgium). Mayer's haematoxylin was used for counterstaining of the slides.

### Total RNA isolation and quantitative RT-PCR

Total RNA was isolated using TRIzol (Invitrogen, Breda, The Netherlands) followed by the RNeasy Mini Protocol (Qiagen, Venlo, The Netherlands). Total RNA (0.2 µg) was reverse transcribed using SuperScript III (Invitrogen) and oligo dT primers (Promega, Madison, USA). Triplicate PCR reactions were performed with 20 pmol of gene-specific primers and Taq DNA polymerase (Promega) using PCR conditions and primers as described previously for *TLRs* [34] and *SPRR2A* [35]. Pre-designed primers and probe mixes for *TLR3*, *CCL5*, *IL1B*, *RNASE7*, *NLRP2*, and *GAPDH* were from Applied Biosystems (Foster City, USA). Threshold cycle numbers (Ct) were determined with 7900HT Fast Real-Time PCR System (Applied Biosystems) and the relative quantities of mRNA per sample were calculated using the ΔΔCt method with *GAPDH* as the calibrator gene. The relative levels of mRNA were determined by setting the mRNA expression level of the lowest expressing control KCs to 1, unless otherwise indicated.

### cRNA synthesis and microarray hybridization

We used four primary KC cultures, HVKp1 and HVKp2 (both vaginal), HFKc1 and ESG2 (both foreskin), as well as four KC cell lines stably maintaining episomal HPV16 or 18, HVK16 (vaginal), HVK18 (vaginal), HCK18 (cervical), and HPV16 (foreskin). Cells were harvested at three conditions: unstimulated, 4 hrs and 24 hrs of 25 µg/ml poly(I:C). Total RNA for these 24 samples was isolated as stated above, and analyzed on an RNA 6000 Nano Lab-on-a-Chip in the 2100 Bioanalyzer (Agilent Technologies, Waldbronn, Germany), showing RIN scores above 9.6. Total RNA (50–100 ng) was reverse-transcribed, amplified and biotin-labeled using the Ambion Illumina TotalPrep RNA Amplification kit (Applied Biosystems, Streetsville, ON, Canada). Concentration measurements were done using the NanoDrop ND-3300 (Isogen Life Science, De Meern, The Netherlands), 750 ng of labeled cRNA was hybridized to Sentrix HumanRef-8 V2 BeadChips (22K, Illumina, San Diego CA, USA), and scanned with BeadArrayer 500GX (Illumina). The samples were randomized for two cRNA synthesis batches and (sub)array location. Raw probe level intensity values were summarized and exported with Illumina probe annotations using Illumina BeadStudio v3.2 (Gene Expression Module BSGX Version 3.2.7). Non-background corrected data were variance stabilizing transformed followed by robust spline normalization [36] using the lumi v1.6.2 [36], [37] and lumiHumanAll.db v1.2.0 [38] BioConductor v2.2 packages in R v2.7.1 (R Development Core Team, www.R-project.org). All microarray data is MIAME compliant and the raw data has been deposited in the MIAME compliant database Gene Expression Omnibus with accession number GSE21260, as detailed on the MGED Society website http://www.mged.org/Workgroups/MIAME/miame.html.

### Analysis of differential gene expression

We fitted a linear model in limma v2.14.7 [39] with ‘virus’ (HPV-positive) and ‘stimulation’ (4 and 24 hrs) effects. We used a nested variable within ‘virus’ for the individual cell lines, where HVKp1 and HVK16 were the reference cells for the HPV-negative and HPV-positive groups, respectively. Multiple-testing corrected p-values [40] and log2 fold changes were extracted for different contrasts. For Table S1, the 4 and 24 hrs timepoints were combined into one F-test in limma. One-dimensional hierarchical clustering of log2 fold changes derived from limma was done in Spotfire DecisionSite 9.1 v19.1.977 using correlation as similarity measure and complete linkage.

### Functional genomics analyses

Functional annotation of the groups of co-regulated genes identified by hierarchical clustering was performed using Anni 2.0 [41]. We used GenMAPP v2.1 [42] to overlay expression on the TLR signaling pathway, which was based on automatic extraction from KEGG [43] hsa04620 (7/17/09) with improved layout using PathVisio v1.0 beta software [44]. The edited pathway is available from GenMAPP and WikiPathways [45].

We used CORE_TF (www.lgtc.nl/CORE_TF) based on TransFac 11.2 and Ensembl 49 [46] to identify over-represented transcription factor binding sites in promoters compared to a random set of 2966 promoters (1000 bp upstream+exon 1). Microarray probe EntrezGene IDs were converted to Ensembl Gene IDs using IDconverter [47], entries resulting in multiple or missing Ensembl Gene IDs were removed. The match cutoff was set to minimize the sum of false positives and false negatives; position weight matrices with a p-value for over-representation ≤0.01 and a frequency below 50% in the random set were selected.

The network was constructed using Ingenuity Pathways Analysis (IPA 7.6; Ingenuity® Systems, Inc., www.ingenuity.com). The 663 HPV signature genes were filtered for the more extreme log fold changes to obtain a gene signature strongly affected by HPVs, and to get the number of genes below 500, which is the maximum limit of IPA for making a network. Genes not connected were deleted, the remaining HPV signature genes that were initially excluded as stated above were included to generate the final network consisting of 212 connected genes. All edges are supported by at least one reference from the literature, from a textbook, or from canonical information stored in the Ingenuity Pathways Knowledge Base.

## Results

### Expression of viral pathogen recognition receptors in KCs

**Figure 1:**
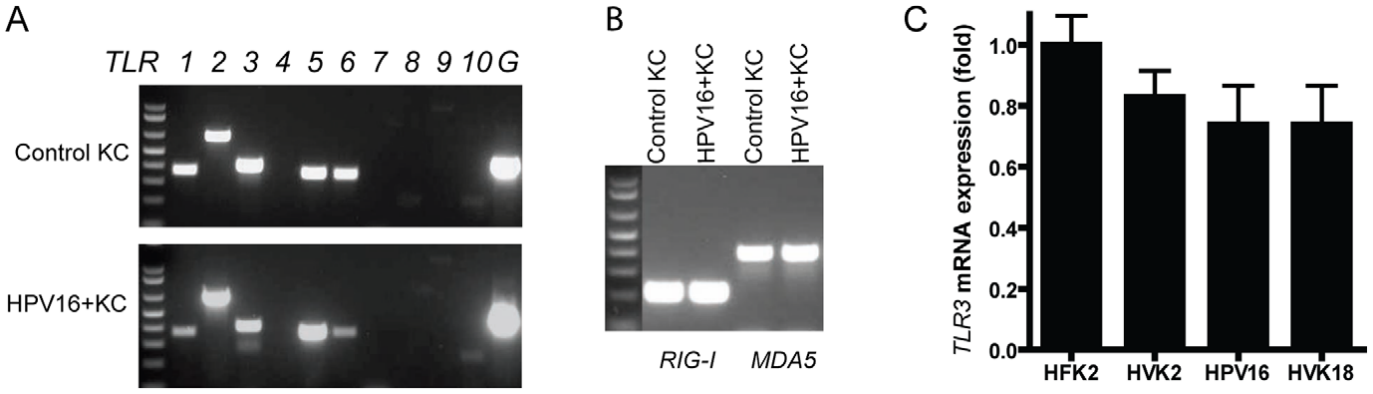
KCs express pathogen recognition receptors. Total RNA of indicated KCs was subjected to RT-PCR (35 cycles) with specific primers for human *TLR1-10*, *GAPDH* (indicated by a G) (A), *RIG-I* or *MDA5* (B). Control KC correspond to HFK2. Size markers (1 kb plus DNA Ladder, Invitrogen) from high to low: 1000, 850, 650, 500, 400, 300, 200, 100 bp; 1.8% agarose gel. (C), TaqMan RT-PCR results showing *TLR3* mRNA expression in HPV-negative (HFK2 and HVK2) and HPV-positive (HPV16 and HVK18) KCs. Fold-changes are relative to HFK2. Data are mean ± SD, n = 3.

We determined the mRNA expression of Toll-like receptors and retinoic acid-inducible gene I (RIG-I)-like receptors in undifferentiated, partially and fully differentiated KCs. Expression of the small proline-rich protein 2A (*SPRR2A*) was used as a molecular marker of KC differentiation (Fig. S1A). Undifferentiated KCs were found to express *TLR1*, *TLR2*, *TLR3*, *TLR5*, *TLR6*, *TLR10*, *RIG-I and MDA5* (Fig. 1A, 1B). Among the viral PRRs, *TLR7*, *TLR8* and *TLR9* were not detectable while *TLR3*, *RIG-I* and *MDA5* were expressed. In parallel experiments, transcripts of *TLR4* and *TLR7-9* were readily detected in mRNA samples from Ramos B-cells and monocytes (Fig. S1B). The expression in KCs is largely in line with previous reports by others [13]. HPV-positive KCs showed essentially the same pattern of PRR expression (Fig. 1A, 1B). Additionally, real-time RT-PCR showed similar levels of *TLR3* in HPV-negative and HPV-positive KCs (Fig. 1C). Upon differentiation KCs also expressed the DNA sensor *TLR9*, which was confirmed by immunohistochemistry in human foreskin and cervical epithelia (Fig. S2). TLR9 was also expressed in the differentiated layers of HPV-positive cervical epithelial neoplasias (Fig. S3). The absence of *TLR4* expression in differentiated KCs, which was confirmed by expression microarray (see below), is consistent with work by others showing that TLR4 was only found in HaCat cells, but not in primary human KCs [16], [48]. The pattern of *TLR* expression in differentiated HPV-positive KCs was similar to that in HPV-negative cells. Thus, HPVs did not affect mRNA expression of the tested PRRs.

### HPV signature genes

We subsequently studied whether HPVs affected the signalling of PRRs using genome-wide expression profiling. Control KCs (n = 4) and KCs with episomal HPV16 or HPV18 genomes (n = 4) of foreskin, vaginal or cervical origin from eight different individuals were used to include biological variation. Since HPVs infect basal KCs, we focused on the viral PRRs expressed in undifferentiated cells, including *TLR3*, *RIG-I* and *MDA5*, which respond to the synthetic dsRNA poly(I:C) [13]. In agreement with the RT-PCR data, the presence of HPV did not change the expression of these PRRs (Table S1).

**Figure 2:**
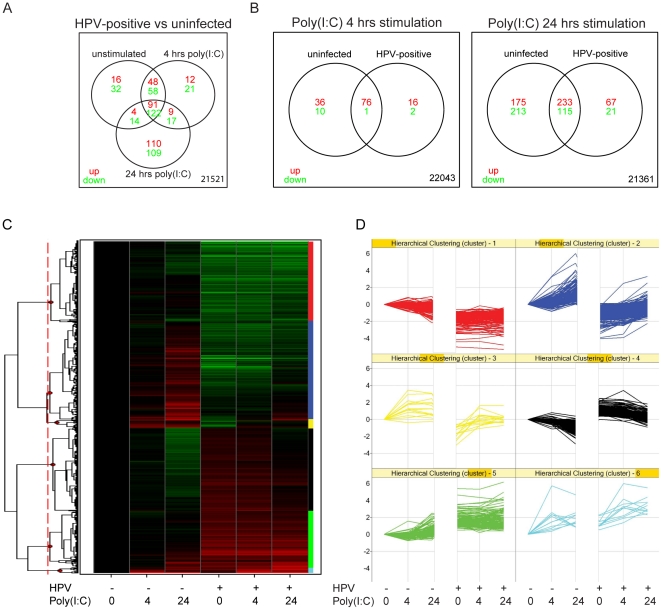
HPVs affect gene expression of KCs both at baseline and upon PRR stimulation. (A), Venn diagram depicting the overlap between 663 HPV signature genes with adjusted *p*-value≤0.05 and absolute log2-fold change≥1 altered by HPVs at baseline (unstimulated) and 4 and 24 hrs of poly(I:C) stimulation. Numbers in red represent upregulated genes while green indicates downregulated genes. (B), Venn diagrams showing the overlapping genes between control and HPV-positive KCs in their response to poly(I:C) stimulation for 4 hrs (left panel) and 24 hrs (right panel). Significance thresholds and colors as in (A). (C), One-dimensional hierarchical clustering of 663 HPV signature genes based on Pearson correlation using a complete linkage algorithm. Rows represent genes, columns represent ordered experimental groups each including four independent biological replicates. Limma log2-fold changes of the indicated conditions compared to the HPV-negative, unstimulated group are shown in the heatmap using red and green for up- and down-regulation, respectively. Black indicates no change. Six clusters based on cutting the gene dendrogram (red dashed vertical line) are indicated with color bars to the right. (D), Profile plots of co-regulated genes grouped according to the six expression clusters. Colors of the gene profiles match the bars to the right of the heatmap in (C). The y-axis shows the log2-fold change compared to HPV-negative, unstimulated KCs, the x-axis shows the ordered sample groups.

To obtain a robust signature of genes affected by HPVs, we selected differentially expressed genes between HPV-positive and -negative KCs at 0, 4 or 24 hrs of poly(I:C) stimulation with a false discovery rate (FDR) of 0.05 (1529 probes). Furthermore, we applied an absolute log2-fold change filter ≥1 to select genes that were at least two-fold up- or downregulated (663 probes representing 634 unique genes), designated “HPV signature genes” (union of genes in Venn diagram Fig. 2A, Table S2). The majority of HPV-specific differentially expressed genes were shared between all three (213) or two (150) conditions, with most overlap between 0 and 4 hrs. Notably, 219 genes were changed in the virus-positive group only after 24 hrs of poly(I:C) stimulation, showing that the effect of HPVs was more pronounced after poly(I:C) stimulation.

### Poly(I:C) response in control KCs

We first focused on the effect of poly(I:C) stimulation in control KCs. While after 4 hrs (Fig. 2B left) we found 123 differentially expressed probes that were mainly upregulated, the response was more balanced and involved over 700 genes after 24 hrs of stimulation (Fig. 2B right). Many genes were upregulated, including pathogen-sensing receptors (*RIG-I*, *MDA5*, *PKR*), adaptor molecules (*MYD88*, *TICAM1/TRIF*, *TICAM2/TRAM*), and interferon regulatory factors (*IRF1*, *IRF6*, *IRF7*), see Table S1. These results are similar to a previous report showing that poly(I:C) stimulation induces antiviral and inflammatory responses in KCs [13]. Overlay of differential expression after 24 hrs of poly(I:C) stimulation on the TLR signaling pathway (KEGG hsa04620) showed upregulation of the Jak-STAT signaling pathway, triggered by temporary upregulation of *IFNB1* after 4 hrs poly(I:C) through the *TRAF3*/*TBK1* signal transduction route, resulting in upregulation of STAT1 and chemotactic cytokines *CXCL10* and *CXCL11*. In addition, via *TRAF6* the NF-kappa-B signaling pathway was triggered, activating cytokines/chemokines *TNF*, *IL1B*, *IL6*, *IL8*, *CCL3*, *CCL4*, and *CCL5* (Fig. S4). The cytoplasmic RNA sensing receptors MDA5 and RIG-I, which are not shown in the TLR signaling pathway, initiate signaling pathways that differ in their initial steps from TLR3 signaling, but converge in the activation of TBK1 and NFKB [13], [49].

### Deregulation of poly(I:C) response in HPV-positive KCs

The differentially expressed genes in the HPV-positive cells upon poly(I:C) stimulation largely overlapped with those in control KCs (Fig. 2B). Next, we studied the effect of the virus in the context of the TLR signaling pathway. Activation of the TLR signaling pathway in HPV-positive KCs upon 24 hrs of poly(I:C) stimulation was largely similar to the response in control cells (Fig. S5). However, when directly comparing HPV-positive and –negative cells after 24 hrs of stimulation, relative downregulation of the adaptor *TICAM1* and several cytokines (*IL1B*, *IL6*, *CCL5/RANTES*) was evident. These results suggest that the dsRNA PRR signaling pathway is less activated in HPV-positive cells (Fig. S6).

### Co-regulated genes downregulated by HPVs

We extended our analyses to the full set of HPV signature genes, and identified genes with similar expression patterns over the sample groups by unsupervised clustering (Fig. 2C, Table S2). The gene dendrogram was cut at six clusters to generate profiles of co-regulated genes (Fig. 2C, 2D). To identify transcription factors possibly involved in the coordinated expression changes, we analyzed the promoter sequences of the genes in each of these clusters for enrichment of predicted transcription factor binding sites [46].

**Figure 3:**
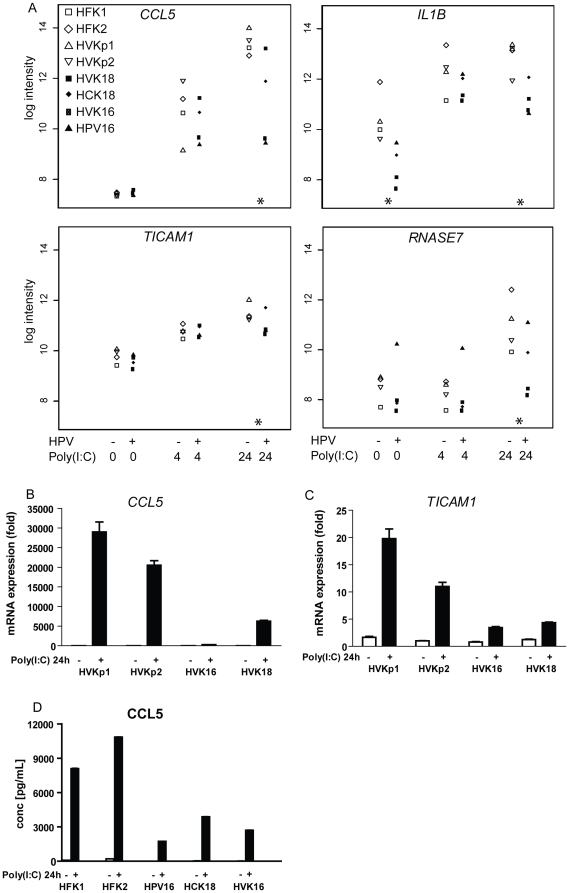
HPVs cause expression changes in immune-related genes. (A), Microarray log2 intensities (y-axis) for the expression levels of four example genes in HPV-negative and HPV-positive KCs, unstimulated or stimulated with poly(I:C) for 4 or 24 hrs. The eight individual KC cultures are color-coded. A star indicates a significant difference between HPV-positive and control KCs (see Materials and Methods for details). TaqMan RT-PCR showing *CCL5* (*RANTES*) (B) and *TICAM1* (C) mRNA expression in control (HVKp1 and HVKp2) and HPV-positive (HVK16 and HVK18) KCs at baseline and after poly(I:C) for 24 hrs. Data are mean ± SD, n = 3. (D), CCL5 secretion of control (HFK1 and HFK2) and HPV-positive (HPV16, HCK18, and HVK16) KCs measured by ELISA. Data are mean ± SD over three replicate samples.

The first three clusters contained genes that were downregulated in HPV-positive compared to HPV-negative cells. Binding sites for early growth response (EGR) family transcription factors, involved in differentiation and mitogenesis, were significantly enriched in these clusters (Table S3). Cluster 1 genes (164 probes), including inflammasome components (*NLRP2*, *PYCARD*), were downregulated in HPV-positive KCs irrespective of poly(I:C) stimulation. Many of these downregulated genes, including several others in expression clusters 2 and 3, are involved in epidermis development and KC differentiation, fitting with the biological effect of HPV in delaying differentiation [50]. Cluster 2 genes (194 probes), including antimicrobials (*DEFB103B*, *LOC728454*, *AQP9*, *RNASE7*, *SRGN*), antigen presenting molecules (HLA-A, -*B*, -*C*, -*G*, *HCP5*), pro-inflammatory cytokines and chemokines (*CCL5/RANTES*, *CSF2/GM-CSF*, *TGF-alpha*, *IL23A*), interferon-inducible genes (*IFI27*, *IFITM1*), and *TICAM1* showed lower expression in the group of unstimulated HPV-positive cells. Moreover, the upregulation of these genes at 24 hrs of poly(I:C) stimulation as found in control KCs was suppressed in HPV-positive cells. Plots with microarray log2 intensities for four probes, *CCL5*/*RANTES*, *IL1B* (cluster 3, see below), *TICAM1* and *RNASE7* show the HPV effect as well as the biological variation inherent to using KCs derived from different individuals and different tissues, combined with two different HPV types (Fig. 3A). Downregulation of *CCL5* and *TICAM1* was confirmed by qRT-PCR (Fig. 3B and 3D), and ELISA showed lower CCL5 secretion in HPV-positive KCs upon poly(I:C) stimulation (Fig. 3C). For the small number of cluster 3 genes (15 probes), including pro-inflammatory cytokines (*IL1B*, *IL1A*, *IL6*), baseline expression (most likely activated by serum components) and upregulation at 4 and 24 hrs of poly(I:C)-stimulation were suppressed in HPV-positive cells. These genes were already upregulated after 4 hrs of stimulation, and showed promoter enrichment of binding sites for Rel/NFKB family members and STAT5 (Table S3).

**Figure 4:**
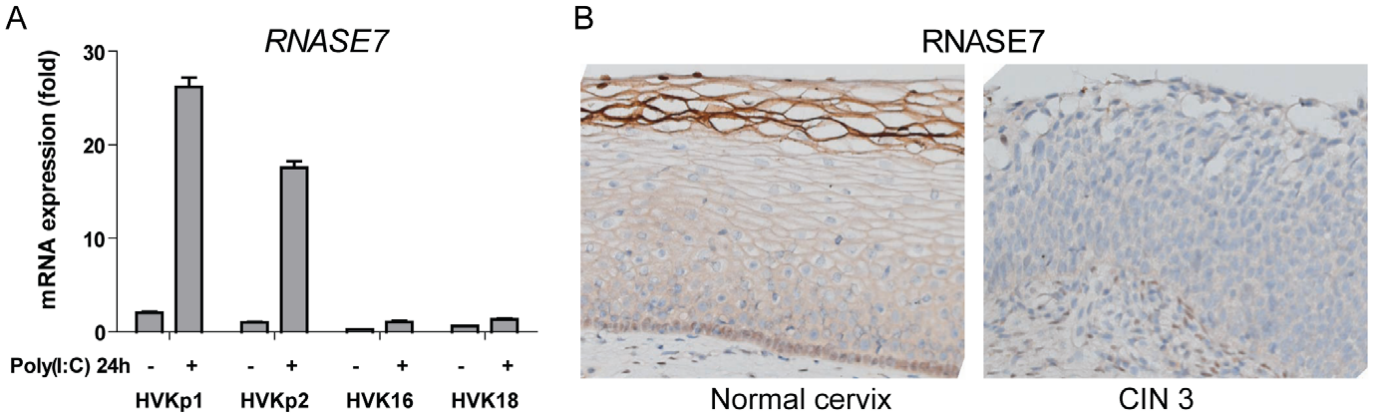
HPV inhibits RNASE7 expression in stimulated KCs and cervical neoplasia. (A), TaqMan RT-PCR showing *RNASE7* mRNA expression in control (HVKp1 and HVKp2) and HPV-positive (HVK16 and HVK18) KCs. Data are mean ± SD, n = 3. (B), RNASE7 protein is downregulated in cervical intraepithelial neoplasia 3 (CIN3). Immunohistochemical staining of paraffin-embedded sections showing RNASE7 protein expression in normal healthy ectocervical epithelium (left) and CIN3 (right). Original magnification 125×. Stainings shown are representative of at least three samples of different individuals.

Interestingly, the majority of expression cluster 2 and 3 genes followed a similar pattern of suppressed poly(I:C) response, suggesting that many of these genes are downstream targets of PRR signaling. We focused on the antimicrobial molecule RNASE7, a member of the RNase A superfamily with broad-spectrum antimicrobial activity and ribonuclease activity [51], [52], which was not known to be affected by viral infection. qRT-PCR confirmed *RNASE7* upregulation upon poly(I:C) stimulation in control KCs, and suppression of poly(I:C)-mediated upregulation in the presence of HPVs (Fig. 4A). Normal cervical epithelial cells expressed RNASE7 throughout the epithelia, and high expression was observed in the basal layer, the *in vivo* equivalent to undifferentiated KCs (Fig. 4B). In contrast, RNASE7 protein was not expressed in any of the layers of undifferentiated cells within a representative HPV-induced CIN3 lesion. These data suggest that by suppressing the gene activation of antimicrobial molecules such as RNASE7, HPVs evaded the innate antiviral responses of the host.

### Co-regulated genes upregulated by HPVs

Clusters 4–6 contained genes that were specifically upregulated in the HPV-positive compared to HPV-negative cells. Cluster 4 genes (167 probes) included heat-shock response genes, cell cycle regulators and genes involved in replication initiation, transcription and splicing. These HPV-activated genes were downregulated upon poly(I:C) stimulation, but not to the same level as in control KCs. Binding sites for MEF2A, involved in the activation of stress-induced genes, and E2F, a family of transcription factors with a crucial role in the control of cell cycle that is indirectly activated by HPV E7, were enriched (Table S3). Cluster 5 (112 probes) contained cancer-related genes including tumor-promoting cytokines/chemokines and their receptors, e.g. *CXCR7*, of which the expression was higher in HPV-positive KCs irrespective of poly(I:C) stimulation. Many transcription factor binding sites were enriched, including motifs binding the oncoprotein MYC (Table S3). Finally, the smallest cluster 6 (11 probes) included several antiviral response genes (*TRIM5*, *ZC3HAV1*, *IFIT2*, *RARRES3*, *CXCL16*) that were stronger upregulated in HPV-positive than in control KCs. Enriched binding sites included IFN-stimulated response element (ISRE), bound by transcription factor ISGF-3, and binding sites bound by interferon-response factors (IRFs).

In summary, the presence of episomal HPVs caused downregulation of genes involved in innate and adaptive immune responses as well as KC differentiation, while upregulated genes were involved in cell cycle, RNA and DNA metabolism. Overall, these data showed that HPVs induced coordinated changes in KC gene expression, detectable in unstimulated ‘baseline’ cells (mainly expression clusters 1, 5, majority of cluster 4) or after poly(I:C) stimulation (mainly expression clusters 2, 3, 6).

### HPVs deregulate cellular networks

**Figure 5:**
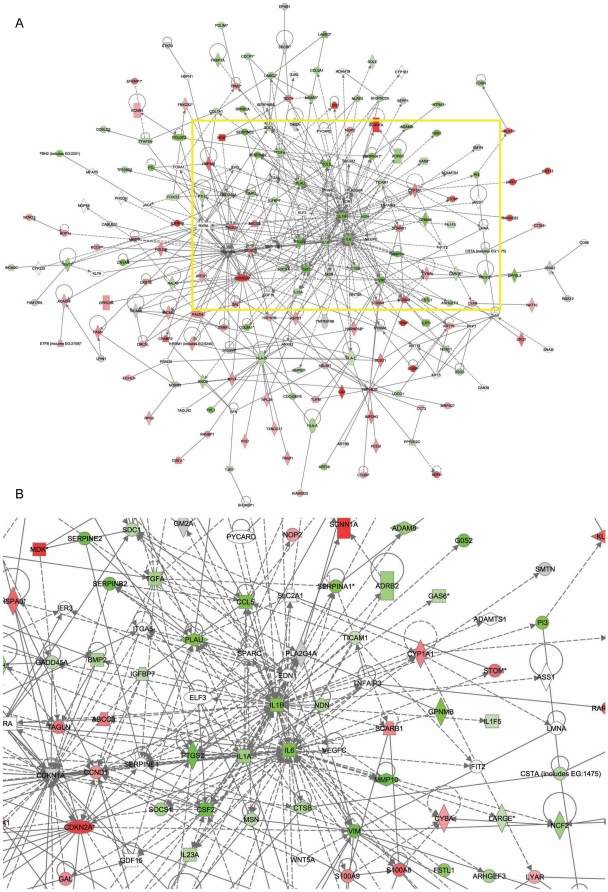
HPVs deregulate a gene network in KCs. A network was constructed of 212 connected HPV signature genes using interaction data curated from literature and high-throughput screens in Ingenuity Pathways Analysis. (A), Overlay with gene expression changes of 24 hrs of poly(I:C)-stimulated HPV-positive KCs versus 24 hrs of poly(I:C)-stimulated HPV-negative KCs. (B), Zoom-in to central region of the network highlighting highly interconnected genes. Molecules are represented as nodes, and the biological relationship between two nodes is represented as an edge (line). Green, downregulated genes; red, upregulated genes; gray, not differentially expressed at the 24-hrs comparison; solid line, direct interaction; dashed line, indirect interaction.

**Figure 6:**
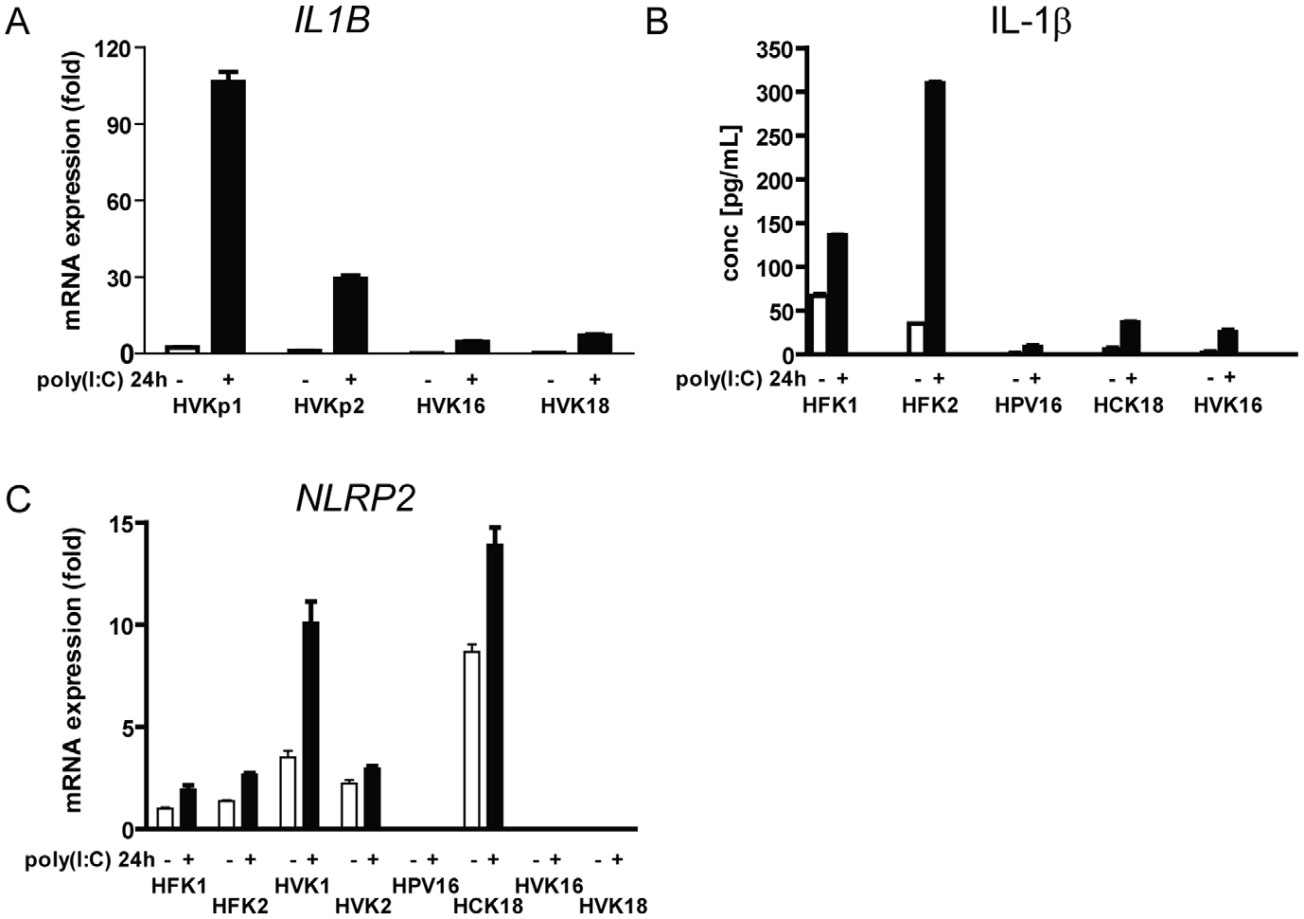
HPVs downregulate IL1B and inflammasome components. (A), TaqMan RT-PCR showing pro-*IL1B* mRNA expression in control (HVKp1 and HVKp2) and HPV-positive (HVK16 and HVK18) KCs. (B), IL-1β protein secretion of control (HFK1 and HFK2) and HPV-positive (HPV16, HCK18 and HVK16) KCs as measured by ELISA. (C), TaqMan RT-PCR showing *NLRP2* mRNA expression in HPV-negative (HFK1, HVK1, HVK2, HFK2) and HPV-positive (HPV16, HCK18, HVK16 and HVK18) KCs. In all three panels, data are mean ± SD, n = 3.

Understanding the network topology of gene and/or protein interactions may identify highly interconnected gene “hubs” targeted by HPVs. Therefore, we explored connections among the HPV signature genes based on literature and high-throughput database information collected in Ingenuity Pathways Analysis [53]. On the resulting network of 212 genes, we overlaid the expression log2-fold changes of HPV-positive versus control KCs after 24 hrs of poly(I:C) stimulation (Fig. 5). The center of the network was formed by the most interconnected gene *IL1B*, necessary for activation of the adaptive immune response [54], and *IL6*. *IL1B* and *IL6* were downregulated, and connected to genes encoding cytokines and antigen presentation molecules that were also lower expressed in HPV-positive cells. We studied *IL1B* in more detail, since it represented a central target for HPV-mediated suppression of both the innate and adaptive immune responses of KCs. RT-PCR data validated the microarray data showing that both the baseline and PRR-stimulated levels of *IL1B* were downregulated in HPV-positive KCs compared to control cells (Fig. 6A). Also, both the baseline and PRR-stimulated IL-1β secretion was lower in HPV-positive KCs (Fig. 6B). Secretion of IL-1β requires activity of both the TLR/NF-kappa-B and the inflammasome pathways [55]. The TLR/NF-kappa-B pathway activates pro-IL-1β expression, which is cleaved to active IL-1β by the inflammasome. In addition to the downregulation of pro-IL-1β, HPVs specifically downregulated the genes encoding inflammasome components *NLRP2* in three of the four HPV-positive lines (Fig. 6C) and *PYCARD*/*ASC*, but not *NALP3*, possibly contributing to the observed lower level of IL-1β. The most interconnected upregulated gene of the network was *CDKN2A*, involved in cell cycle progression. Thus, by targeting highly interconnected genes, HPVs reprogrammed the gene network of KCs in favor of immune escape and cell proliferation of HPV-positive cells.

## Discussion

We studied systematic differences in genome-wide expression profiles of control and HPV-positive undifferentiated (basal) KCs focusing on immune-related effects. The parallel analysis of several control and HPV16- and 18-positive KCs from several genital tissues ensured that the results can be generalized. The HPV-positive KCs expressed the full array of HPV genes and mimic latent HPV infection *in vivo*, which is also reflected by the fact that these cells display the entire differentiation-dependent HPV life cycle upon culture in organotypic raft cultures [28], [29]. Our studies revealed that while KCs are well equipped to respond to viral pathogens, latent infection with HPV results in suppression downstream of the PRRs as reflected by lower expression levels of effector molecules involved in innate and adaptive immune response.

No difference was observed in expression levels of viral RNA PRRs *TLR3*, *TLR9*, *RIG-I*, *MDA5* and *PKR* between control and HPV-positive KCs. We found that viral DNA PRR TLR9 was lacking in the basal layers in stratified squamous epithelia, but expressed in the suprabasal layers of the non-neoplastic epithelium. Previous studies suggested that E6/E7 expression affected neither the expression nor the function of TLR9 [17], whereas others reported that E6/E7 expression resulted in loss of TLR9 expression [12]. Our data showed that forced differentiation of HPV-positive KCs resulted in the expression of TLR9, however, as HPVs inhibits differentiation this may appear as TLR9 loss similar to what was seen previously [12]. Thus, TLR9 is absent in the cells targeted by HPV, but other viral PRRs are expressed, including RIG-I that has been shown to indirectly function as a PRR for DNA viruses [30]–[32], suggesting that in essence undifferentiated KCs can sense HPV infection.

As there were no overt differences in the expression levels of PRRs, we focused on the interference of HPVs with the downstream pathogen-sensing machinery. First, our data showed that HPVs downregulated genes that have a direct antimicrobial function. Moreover, the presence of HPVs was associated with the downregulation of an array of pro-inflammatory and chemotactic cytokines, and antigen-processing and presenting molecules, and IL-1β and IL6 were the hubs in the center of this HPV signature gene network. Notably, the expression level of most of these genes was already lower at baseline. Poly(I:C), which triggers viral PRRs including TLR3 and importantly also RIG-I, increased their expression level in HPV-positive KCs albeit not to the same level as in control KCs. Previously it was shown that HPV31-positive KCs responded less well to interferon stimulation [27] and this fits with our own data showing that interferon-inducible genes (cluster 2) are downregulated. Apparently, this is not the only immune signaling pathway that is downregulated by HPV as our data reveal that also the TLR and the RIG-I-like receptor signaling pathways are suppressed in HPV-positive KCs. Notably, the failure of HPV31-positive KCs to respond to interferon was associated with downregulation of STAT1 (25). Specific downregulation of STAT1 was found only in our HPV16-positive KCs (data not shown) suggesting that there may be a number of type-specific interactions with the host's immune system. Together these data suggest that HPVs dampen but do not block PRR signaling, and imply that the attraction of innate immune cells to the site of HPV infection, the subsequent initiation of adaptive immunity as well as the recognition of HPV-infected KCs is slowed down but not prevented. This clearly corresponds with the fact that it may take months or even a year to control HPV infections [4], and the increase in HPV-infected subjects capable of mounting an HPV-specific immune response in time [56]. Furthermore, it fits with the detection of HPV-specific memory responses after infection [9], [57], [58].

In particular, we found that HPVs downregulated toll-like receptor adaptor molecule 1 (*TICAM1*), a critical molecule in the TLR3 pathway that mediates NF-kappa-B and interferon-regulatory factor (IRF) activation via downstream molecules TRAF3, TRAF6 and RIP1 [59]. Notably, the other poly(I:C) recognizing PRRs also malfunction in HPV-positive KCs suggesting that HPVs affect the TBK1 and NF-kappa-B signaling pathways downstream of the PRRs and implying that downregulation of *TICAM1* is just part of the immune evasion strategy of HPVs. This is also illustrated by our finding that HPVs downregulated inflammasome components – needed to convert pro-IL-1β to the active form of IL-1β [60] - contributing to the lower secretion of IL-1β by HPV-positive cells. Of all candidate downstream targets IRF1 [25], IRF3 [24], the coactivator CPB [61], the IkB kinase complex [62], and the interferon-stimulated gene factor 3 (ISGF3) transcription complex [63] have been named as targets for either E6 and E7 proteins of HPV responsible for downregulating NF-kappa-B and TBK1 signaling. Others, however, have shown that E6 – instead of downregulating - may promote NF-kappa-B signaling [26], [64]. Importantly, all of these studies relied on the overexpression of either one or both oncoproteins, which is more relevant for our understanding of HPV-transformed cells. The strength of our study lies in the use of KCs with episomal expression of the full array of HPV genes reflecting latent infection [28], [29]. It would be of great importance to perform a genome-wide study of HPV-positive KCs during differentiation and interaction with (innate) immune cells thereby closely mimicking the situation *in situ*, but such an experiment would be technically challenging.

Non-cleared infection with high-risk HPVs leads to cervical and other anogenital carcinomas in which the virus genome integrates in the host genome [2], . The replication cycle of the virus is tightly coupled to the differentiation of basal KCs to stratified squamous epithelia and it is well known that HPVs inhibit KC differentiation [11]. In our expression data, this was reflected by concerted upregulation of cell cycle regulators and DNA/RNA synthesis, and downregulation of epidermis development and KC differentiation genes. *CDKN2A*, a critical cell cycle regulator upregulated by HPVs, was identified as one of the highly-connected hub genes in the network of HPV signature genes. Similar results were described by Nees et al. using a cDNA oncochip [26].

We have shown that HPV16 and 18 dampen a cellular immune-related network in HPV-positive KCs, and affect a much broader spectrum of PRR responses than the previously described IRF route. Our study provides a framework for future exploration into the molecular mechanisms involved in HPV-downregulated immunity. The biological variation in gene expression between different donors might reflect genomic variation that could play a role the balance between clearance and persistence of HPV. Additionally, it would be of interest to study if other viruses capable of causing persistent infection or low-risk HPVs that cause benign genital warts use similar mechanisms to escape host's immune responses.

## Supporting Information

Figure S1 **Positive controls for keratinocyte differentiation and PRR expression.** (A), Reverse transcription PCR detection of the small proline-rich protein 2A (SPRR2A), a molecular marker of KC differentiation after 20, 25 and 30 PCR cycles in undifferentiated (1), partially differentiated (2) and fully differentiated (3) normal foreskin keratinocytes. SPRR2A expression was absent from undifferentiated KCs, low in Ca2+-treated KCs and high in KCs cultured in suspension with Ca2+ and methylcellulose, confirming that the KCs consisted of undifferentiated (basal) cells and differentiated in vitro. (B), Reverse transcription PCR detection of TLRs 1–10 and GAPDH (“G”) in mRNA samples from Ramos B-cells and monocytes.
(PDF)

Figure S2 TLR9 expression in stratified squamous epithelia progressively increases with KC differentiation stage. (A), Total RNA of the indicated cells was subjected to RT-PCR (35 cycles) with specific primers human TLR1–10 or GAPDH as indicated by a “G”. (B), TaqMan real-time PCR was performed for TLR9 on total RNA samples from indicated cell types. TLR9 expression was normalized against GAPDH mRNA levels. Data represent an average of three independent experiments. (C), Immunohistochemical staining of paraffin-embedded healthy foreskin sections and (D) sections of healthy ectocervical epithelium with human TLR9-specific monoclonal antibody (left panels) or isotype control antibody (right panels) in combination with peroxidase-conjugated secondary antibody. Cell nuclei were counterstained with haematoxylin. Original magnification 125×. Stainings shown are representative of at least three samples of different origin.
(PDF)

Figure S3 TLR9 is expressed in differentiated cell layers of HPV-positive cervical epithelial neoplasia. Immunohistochemical staining with TLR9-specific or isotype control antibody of paraffin-embedded sections of normal and dysplastic genital epithelia. Staining was performed as described in the legend to Figure S2. Original magnification 125×. Sections of the following epithelial samples are shown: A) normal cervical epithelium, B) CIN1, C) CIN2.
(PDF)

Figure S4 TLR signalling in KCs. Toll-like receptor signalling pathway (KEGG hsa4620) overlaid with differentially expressed genes between 24 hrs poly(I:C) stimulated and unstimulated uninfected keratinocyte cultures. Differentially expressed genes (FDR≤0.05) were colored bright red (log2 fold change≥1) or dim red (log2 fold change between 0 and 1) for upregulation upon poly(I:C) stimulation, or bright green (log2 fold change≤−1) or dim green (log2 fold change between 0 and −1) for downregulation. Grey boxes represent genes not fulfilling the above criteria, while white boxes are genes not represented by probes on the array.
(PDF)

Figure S5 TLR signalling in HPV-KCs. Toll-like receptor signalling pathway (KEGG hsa4620) overlaid with differentially expressed genes between 24 hrs poly(I:C) stimulated and unstimulated HPV-infected keratinocyte cultures. For explanation of colors, see Figure S4.
(PDF)

Figure S6 Differential TLR signalling between HPV-KCs and KCs. Toll-like receptor signalling pathway (KEGG hsa4620) overlaid with differentially expressed genes between HPV-infected and uninfected keratinocytes, both after 24 hrs poly(I:C) stimulation. Differentially expressed genes (FDR≤0.05) were colored according to their log2 fold change (see legend Figure S4) for upregulation (red) or downregulation (green) in HPV-positive cells.
(PDF)

Table S1 Differential expression of pattern recognition receptors and signalling molecules in HPV-infected and uninfected keratinocytes.
(PDF)

Table S2 HPV signature genes.
(XLS)

Table S3 Enrichment of transcription factor binding sites in HPV signature gene promoters.
(PDF)
