# Supplementary figures and images for: Human Papillomavirus Deregulates the Response of a Cellular Network Comprising of Chemotactic and Proinflammatory Genes
Source: PLoS One. 2011 Mar 14;6(3):e17848. doi: 10.1371/journal.pone.0017848 (PMC3056770; doi:10.1371/journal.pone.0017848)

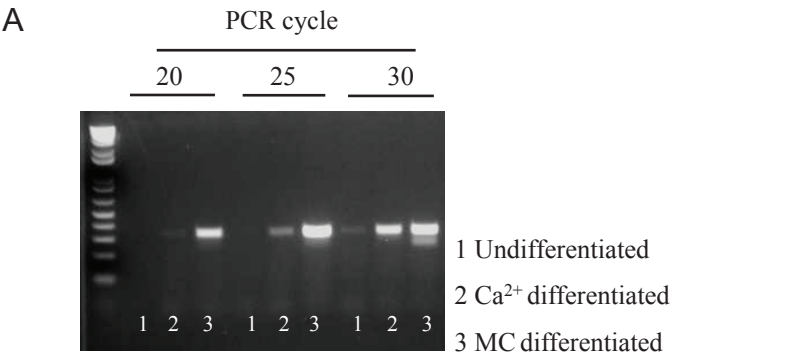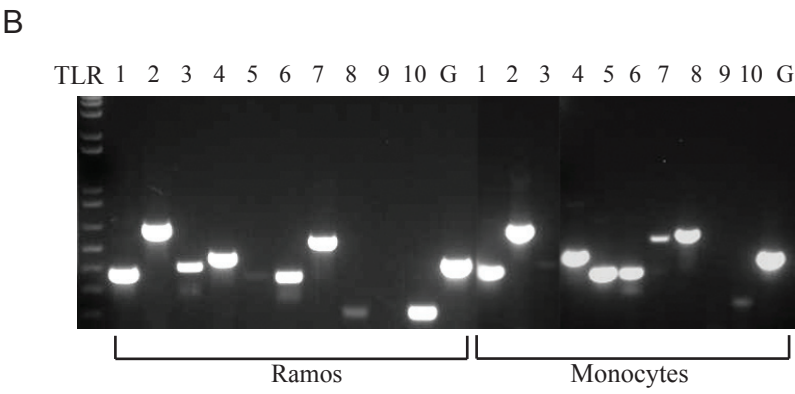

Supplement: Figure S1 — Positive controls for keratinocyte differentiation and PRR expression. (A), Reverse transcription PCR detection of the small proline-rich protein 2A (SPRR2A), a molecular marker of KC differentiation after 20, 25 and 30 PCR cycles in undifferentiated (1), partially differentiated (2) and fully differentiated (3) normal foreskin keratinocytes. SPRR2A expression was absent from undifferentiated KCs, low in Ca2+-treated KCs and high in KCs cultured in suspension with Ca2+ and methylcellulose, confirming that the KCs consisted of undifferentiated (basal) cells and differentiated in vitro. (B), Reverse transcription PCR detection of TLRs 1–10 and GAPDH (“G”) in mRNA samples from Ramos B-cells and monocytes. (PDF) [file pone.0017848.s001.pdf]

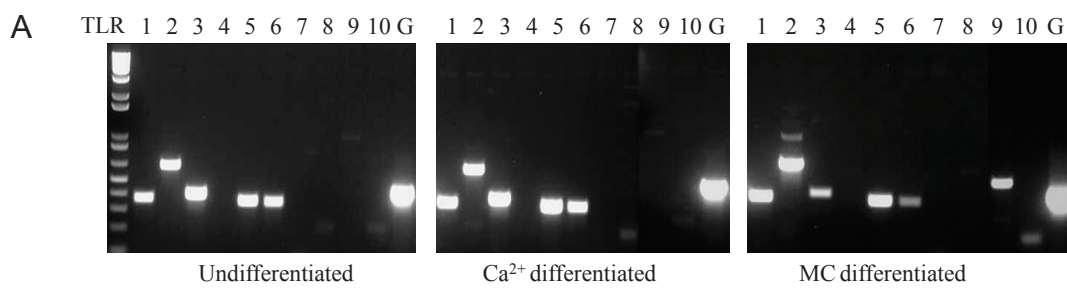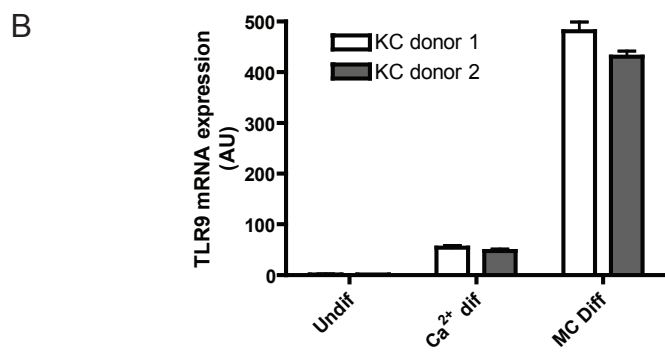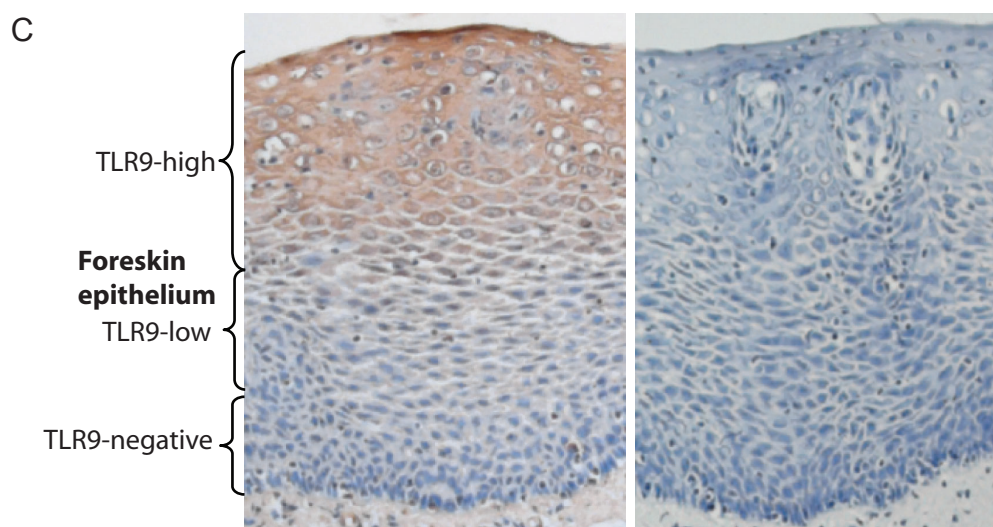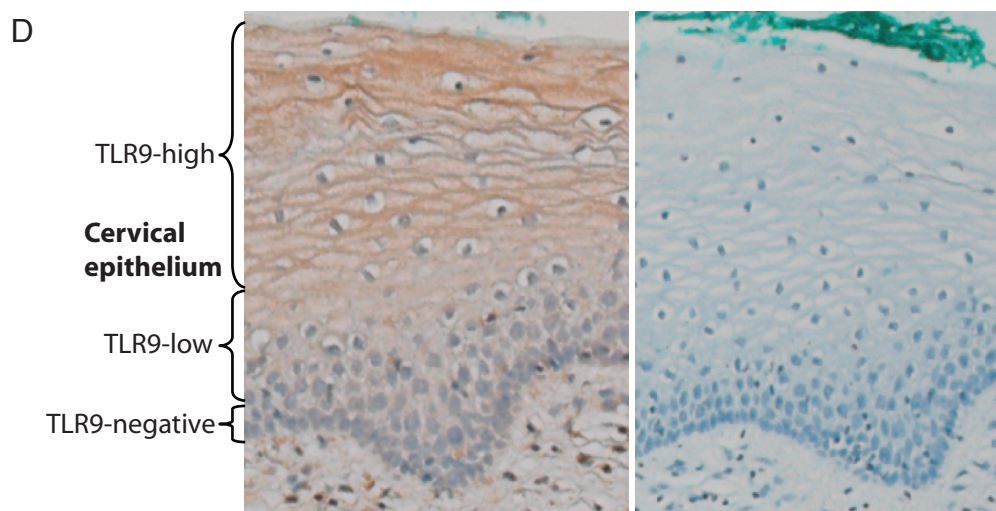

Supplement: Figure S2 — TLR9 expression in stratified squamous epithelia progressively increases with KC differentiation stage. (A), Total RNA of the indicated cells was subjected to RT-PCR (35 cycles) with specific primers human TLR1–10 or GAPDH as indicated by a “G”. (B), TaqMan real-time PCR was performed for TLR9 on total RNA samples from indicated cell types. TLR9 expression was normalized against GAPDH mRNA levels. Data represent an average of three independent experiments. (C), Immunohistochemical staining of paraffin-embedded healthy foreskin sections and (D) sections of healthy ectocervical epithelium with human TLR9-specific monoclonal antibody (left panels) or isotype control antibody (right panels) in combination with peroxidase-conjugated secondary antibody. Cell nuclei were counterstained with haematoxylin. Original magnification 125×. Stainings shown are representative of at least three samples of different origin. (PDF) [file pone.0017848.s002.pdf]

Normal

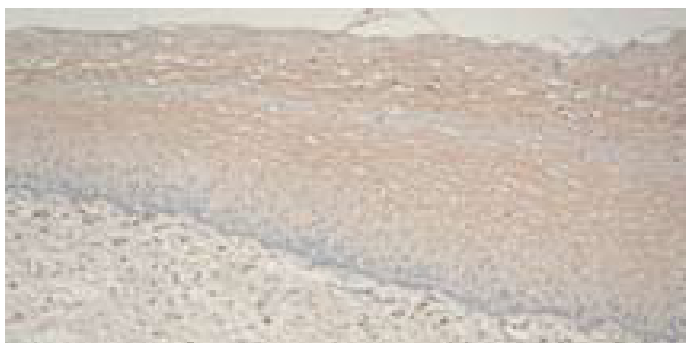

TLR9

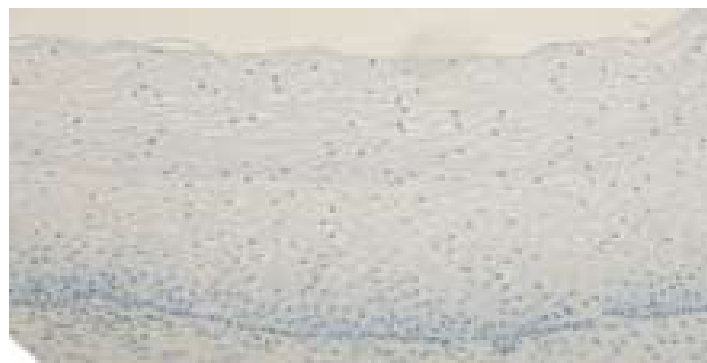

Isotype

CIN1

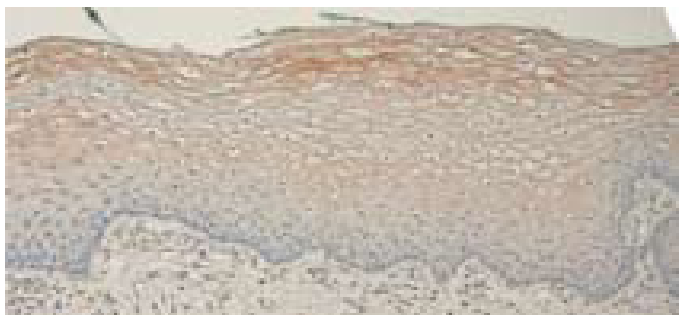

TLR9

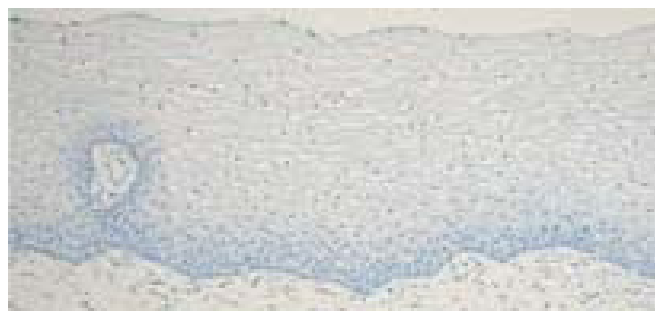

Isotype

CIN2

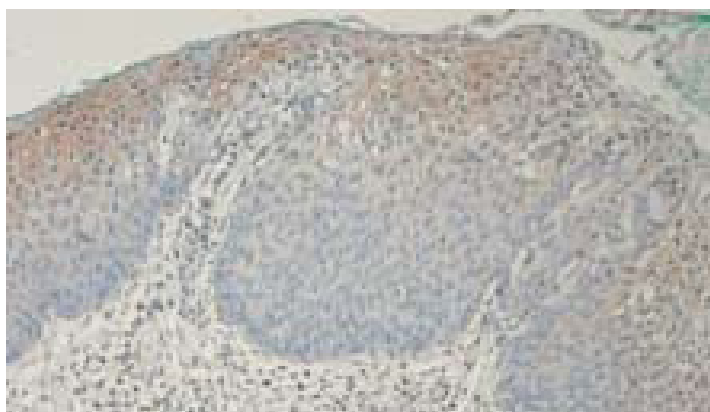

TLR9

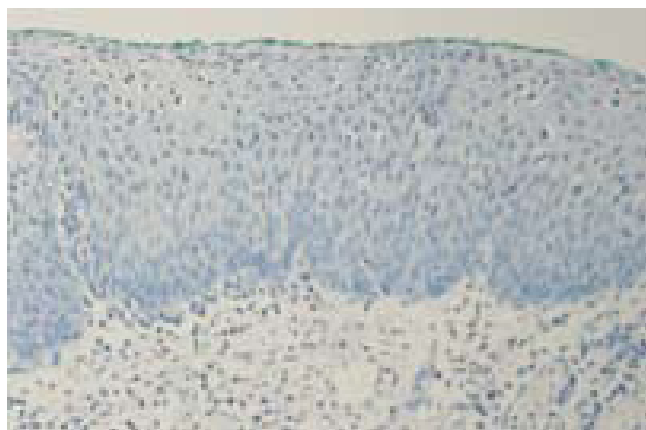

Isotype

Supplement: Figure S3 — TLR9 is expressed in differentiated cell layers of HPV-positive cervical epithelial neoplasia. Immunohistochemical staining with TLR9-specific or isotype control antibody of paraffin-embedded sections of normal and dysplastic genital epithelia. Staining was performed as described in the legend to Figure S2. Original magnification 125×. Sections of the following epithelial samples are shown: A) normal cervical epithelium, B) CIN1, C) CIN2. (PDF) [file pone.0017848.s003.pdf]
