# Supplementary material for: Human Papillomavirus Deregulates the Response of a Cellular Network Comprising of Chemotactic and Proinflammatory Genes
Source: PLoS One. 2011 Mar 14;6(3):e17848. doi: 10.1371/journal.pone.0017848 (PMC3056770; doi:10.1371/journal.pone.0017848)

TLR signaling pathway: 24h polyI:C stimulated vs unstimulated uninfected keratinocytes

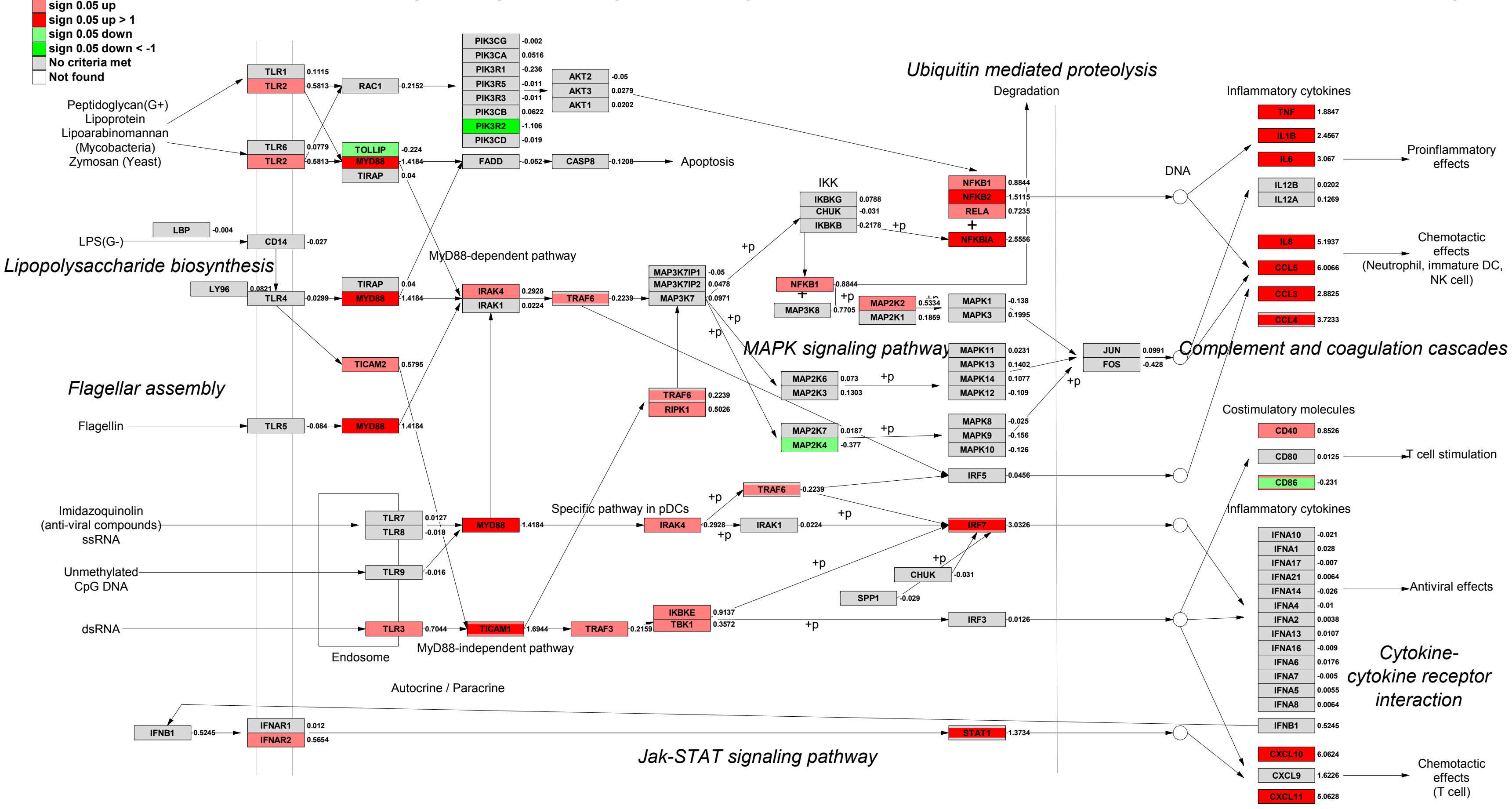

Supplement: Figure S4 — TLR signalling in KCs. Toll-like receptor signalling pathway (KEGG hsa4620) overlaid with differentially expressed genes between 24 hrs poly(I:C) stimulated and unstimulated uninfected keratinocyte cultures. Differentially expressed genes (FDR≤0.05) were colored bright red (log2 fold change≥1) or dim red (log2 fold change between 0 and 1) for upregulation upon poly(I:C) stimulation, or bright green (log2 fold change≤−1) or dim green (log2 fold change between 0 and −1) for downregulation. Grey boxes represent genes not fulfilling the above criteria, while white boxes are genes not represented by probes on the array. (PDF) [file pone.0017848.s004.pdf]
