# Supplementary material for: Human Papillomavirus Deregulates the Response of a Cellular Network Comprising of Chemotactic and Proinflammatory Genes
Source: PLoS One. 2011 Mar 14;6(3):e17848. doi: 10.1371/journal.pone.0017848 (PMC3056770; doi:10.1371/journal.pone.0017848)

TLR signaling pathway: 24h polyI:C stimulated vs unstimulated HPV-positive keratinocytes

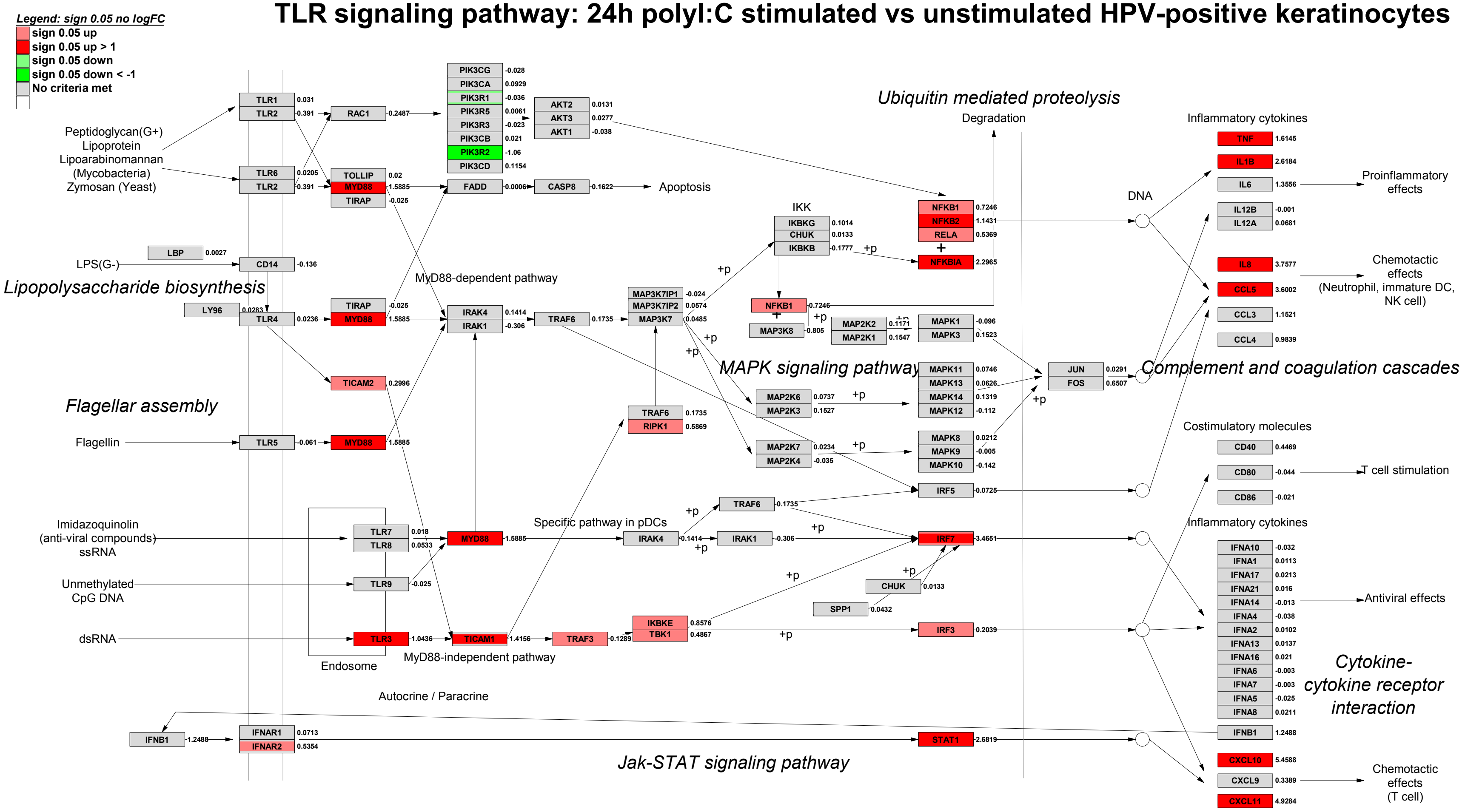

Supplement: Figure S5 — TLR signalling in HPV-KCs. Toll-like receptor signalling pathway (KEGG hsa4620) overlaid with differentially expressed genes between 24 hrs poly(I:C) stimulated and unstimulated HPV-infected keratinocyte cultures. For explanation of colors, see Figure S4. (PDF) [file pone.0017848.s005.pdf]
