# Supplementary material for: Human Papillomavirus Deregulates the Response of a Cellular Network Comprising of Chemotactic and Proinflammatory Genes
Source: PLoS One. 2011 Mar 14;6(3):e17848. doi: 10.1371/journal.pone.0017848 (PMC3056770; doi:10.1371/journal.pone.0017848)

TLR signaling pathway: 24h polyI:C stimulated HPV-positive vs 24h polyI:C stimulated uninfected keratinocytes

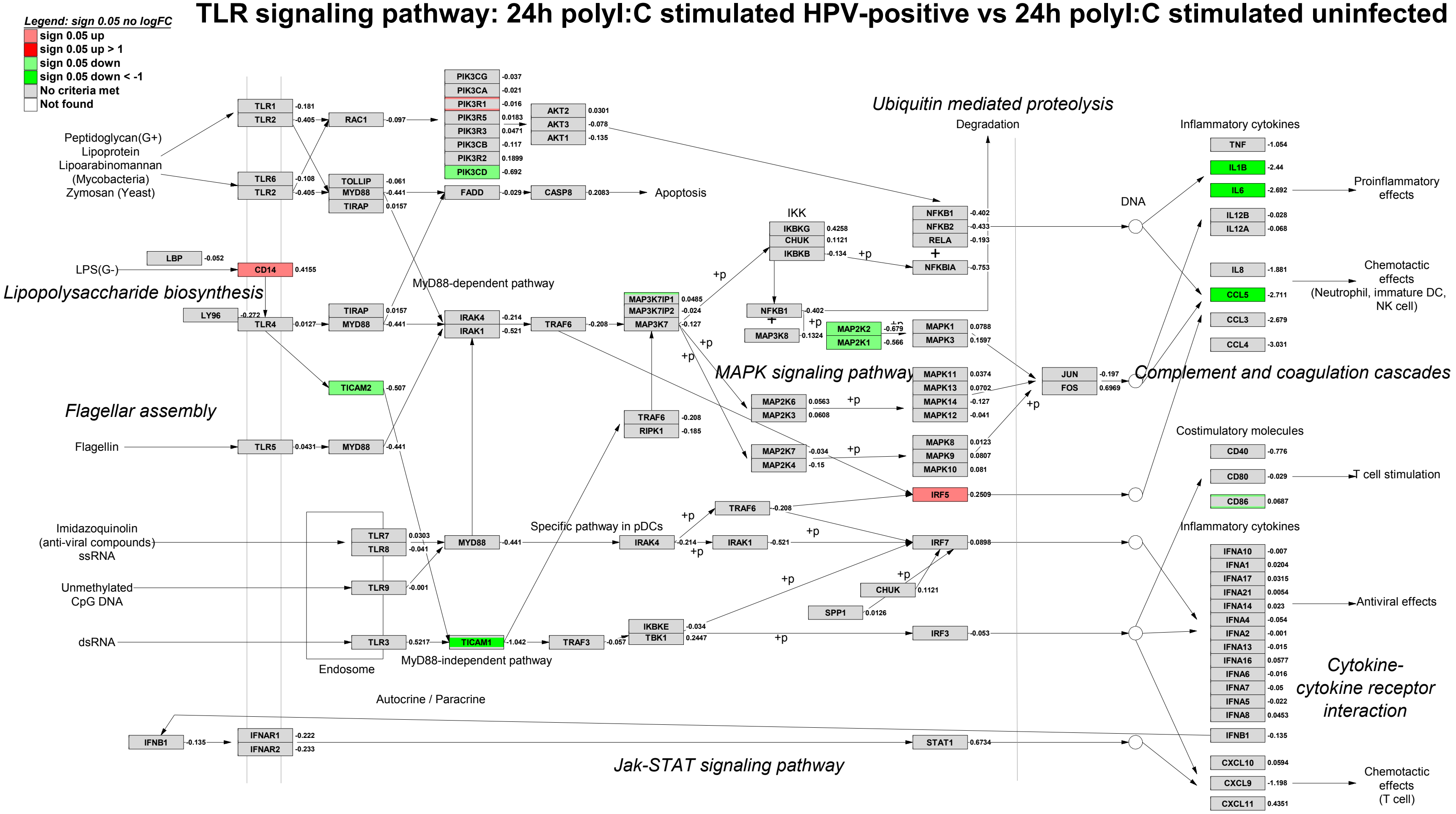

Supplement: Figure S6 — Differential TLR signalling between HPV-KCs and KCs. Toll-like receptor signalling pathway (KEGG hsa4620) overlaid with differentially expressed genes between HPV-infected and uninfected keratinocytes, both after 24 hrs poly(I:C) stimulation. Differentially expressed genes (FDR≤0.05) were colored according to their log2 fold change (see legend Figure S4) for upregulation (red) or downregulation (green) in HPV-positive cells. (PDF) [file pone.0017848.s006.pdf]
