# Supplementary material for: Human Papillomavirus Deregulates the Response of a Cellular Network Comprising of Chemotactic and Proinflammatory Genes
Source: PLoS One. 2011 Mar 14;6(3):e17848. doi: 10.1371/journal.pone.0017848 (PMC3056770; doi:10.1371/journal.pone.0017848)
Supplement: Table S1 — Differential expression of pattern recognition receptors and signalling molecules in HPV-infected and uninfected keratinocytes. (PDF) [file pone.0017848.s007.pdf]

Supplementary Table S1.
Differential expression of pattern recognition receptors and signalling molecules in HPV-infected and uninfected keratinocytes.

		**HPV-infected versus Uninfected***				**PolyI:C Stimulation****		
**Gene Symbol**	**ProbeID**	**Unstimulated**	**4 hrs PolyI:C**	**24 hrs PolyI:C**	**Change*****	**Uninfected**	**HPV-infected**	**Change**
**TLRs**								
TLR1	ILMN_1731048	0.5075	0.8101	0.1245		0.0639	0.9252	
TLR2	ILMN_1772387	0.6670	0.7159	0.2514		**0.0116**	0.1590	**up**
TLR3	ILMN_1689578	0.8196	0.9781	0.2745		**0.0179**	**0.0075**	**up**
TLR4	ILMN_1706217	0.8120	0.8130	0.8617		0.4416	0.6560	
TLR5	ILMN_1722981	0.9297	0.9036	0.7692		0.3101	0.5266	
TLR6	ILMN_1749287	0.8026	0.9457	0.4102		0.2668	0.9707	
TLR7	ILMN_1677827	0.7841	0.7538	0.6670		0.1113	0.8872	
TLR8	ILMN_1682251	0.5907	0.6089	0.5755		0.4787	0.1055	
TLR8	ILMN_1657892	0.9512	0.6378	0.6467		0.8404	0.4786	
TLR8	ILMN_1705047	0.9912	0.9477	0.9704		0.8519	0.7386	
TLR9	ILMN_1679798	0.9517	0.9791	0.9929		0.8768	0.7921	
TLR10	ILMN_1719905	0.2354	0.7753	0.5113		0.6290	0.9121	
**Virus PRRs**								
DDX58/RIG-I	ILMN_1797001	0.9137	0.8457	0.5615		**0.0002**	**0.0004**	**up**
IFIH1/MDA5	ILMN_1781373	0.8513	0.9743	0.9656		**0.0001**	**0.0001**	**up**
EIF2AK2/PKR	ILMN_1706502	0.3856	0.4664	0.8941		0.0970	**0.0128**	**up**
NLRP3	ILMN_1712026	0.1002	**0.0261**	**0.0199**	down	0.0620	0.4713	
NLRP3	ILMN_1713379	0.8305	0.1922	0.5545		0.5681	0.6947	
**Adaptors**								
MYD88	ILMN_1738523	0.4745	0.4618	0.6085		**0.0061**	**0.0071**	**up**
TICAM1/TRIF	ILMN_1724863	0.0865	0.2568	**0.0178**	**down**	**0.0001**	**0.0006**	**up**
TICAM1/TRIF	ILMN_1815079	0.8277	0.6189	0.4977		0.0595	0.2895	
TICAM2/TRAM	ILMN_1651346	0.3780	0.3253	**0.0209**	down	**0.0007**	0.0664	up
**IRFs**								
IRF1	ILMN_1708375	0.5398	0.7765	0.9370		**0.0000**	**0.0002**	**up**
IRF2	ILMN_1765547	0.9592	0.5474	0.8788		0.1001	0.8366	
IRF3	ILMN_1765649	0.0521	0.3810	0.7662		0.8172	**0.0332**	up
IRF4	ILMN_1754507	0.5804	0.9340	0.9360		0.9231	0.6536	
IRF5	ILMN_1670576	**0.0358**	**0.0145**	**0.0179**	up	0.7228	0.5558	
IRF6	ILMN_1725946	0.0830	0.1206	0.0761		**0.0023**	**0.0053**	**up**
IRF7	ILMN_1674646	0.4028	0.2741	0.3775		**0.0001**	**0.0001**	**up**
IRF7	ILMN_1798181	0.9638	0.9692	0.7348		**0.0140**	**0.0002**	**up**
IRF8	ILMN_1666594	0.9293	0.9970	0.8020		0.8348	0.8932	

* P-values were calculated using a linear model (Smyth, 2004) and adjusted for multiple testing according to Benjamini and Hochberg (1995). P-values below 0.05 are in bold.
** The 4 hrs versus unstimulated and 24 hrs versus unstimulated comparisons were combined into one F-test using limma.
*** Change indicates direction of expression change. Changes that exceed the arbitrary biological significance threshold of logFC 1are in bold.
