# Supplementary material for: Human Papillomavirus Deregulates the Response of a Cellular Network Comprising of Chemotactic and Proinflammatory Genes
Source: PLoS One. 2011 Mar 14;6(3):e17848. doi: 10.1371/journal.pone.0017848 (PMC3056770; doi:10.1371/journal.pone.0017848)
Supplement: Table S3 — Enrichment of transcription factor binding sites in HPV signature gene promoters. (PDF) [file pone.0017848.s009.pdf]

Supplementary Table S3.
Enrichment of transcription factor binding sites in HPV signature gene promoters.

A. Expression Clusters 1-3, downregulated in HPV-positive keratinocytes

Expression Cluster 1 (115 promoters)

matrix*	# exp**	freq exp	freq ran***
**AHR_Q5**	6	0.0522	0.0182
AP1_Q2_01	17	0.1478	0.0819
AP1_Q6	21	0.1826	0.1062
AP2_Q3	74	0.6435	0.4440
AP2_Q6	60	0.5217	0.3439
AP2ALPHA_02	43	0.3739	0.2569
**AP2GAMMA_01**	46	0.4000	0.3007
**BACH2_01**	6	0.0522	0.0061
CACD_01	71	0.6174	0.4872
**CBF_01**	26	0.2261	0.1396
**DEC_Q1**	8	0.0696	0.0307
EGR_Q6	18	0.1565	0.0860
**EGR1_01**	31	0.2696	0.1790
ETF_Q6	61	0.5304	0.3982
**ETS_Q6**	28	0.2435	0.1571
**ETS1_B**	42	0.3652	0.2653
**EVI1_01**	3	0.0261	0.0071
GC_01	75	0.6522	0.3941
**HEN1_01**	1	0.0087	0.0003
**HIC1_02**	24	0.2087	0.1335
**HMX1_01**	2	0.0174	0.0013
**HNF4_01**	9	0.0783	0.0354
KROX_Q6	37	0.3217	0.1800
LRF_Q2	45	0.3913	0.2471
MAZ_Q6	49	0.4261	0.2980
MUSCLE_INI_B	47	0.4087	0.2933
MZF1_02	45	0.3913	0.2873
**NRSF_01**	8	0.0696	0.0310
PAX5_01	73	0.6348	0.4673
POLY_C	1	0.0087	0.0003
SP1_Q2_01	48	0.4174	0.2687
SP1_Q4_01	64	0.5565	0.3186
SP1_Q6	61	0.5304	0.3007
SP1_Q6_01	63	0.5478	0.3109
SP3_Q3	40	0.3478	0.2421
**SREBP1_Q6**	43	0.3739	0.2751
**WT1_Q6**	34	0.2957	0.1831

Expression Cluster 2 (153 promoters)

matrix	# exp	freq exp	freq ran
AP1_Q2_01	22	0.1438	0.0819
**AP1_Q4_01**	12	0.0784	0.0293
AP1_Q6	26	0.1699	0.1062
**AP1_Q6_01**	20	0.1307	0.0681
AP2_Q3	87	0.5686	0.4440
AP2_Q6_01	74	0.4837	0.3560
CACD_01	89	0.5817	0.4872
EGR_Q6	23	0.1503	0.0860
GC_01	77	0.5033	0.3941
**HIC1_03**	33	0.2157	0.1463
HSF1_Q6	1	0.0065	0.0003
KROX_Q6	41	0.2680	0.1800
**NFY_01**	16	0.1046	0.0583
**NGFIC_01**	46	0.3007	0.2023
SP1_Q2_01	55	0.3595	0.2687
SP1_Q4_01	62	0.4052	0.3186
SP1_Q6	61	0.3987	0.3007
SP1_Q6_01	68	0.4444	0.3109
**ZNF219_01**	6	0.0392	0.0152

Expression Cluster 3 (12 promoters)

matrix	# exp	freq exp	freq ran
AP2ALPHA_01	5	0.4167	0.1618
**CEBP_Q2_01**	7	0.5833	0.2357
**CREL_01**	3	0.2500	0.0738
**DBP_Q6**	5	0.4167	0.1581
**E2_Q6_01**	1	0.0833	0.0051
**EGR3_01**	7	0.5833	0.2610
**GFI1B_01**	3	0.2500	0.0738
**HP1SITEFACTOR_Q6**	1	0.0833	0.0115
HSF2_01	2	0.1667	0.0260
**IK1_01**	5	0.4167	0.1642
**NFKAPPAB65_01**	2	0.1667	0.0266
**PITX2_Q2**	2	0.1667	0.0243
**RREB1_01**	1	0.0833	0.0040
**SMAD_Q6**	6	0.5000	0.2073
**SREBP1_02**	8	0.6667	0.3645
**STAT5A_01**	2	0.1667	0.0361

B. Expression Clusters 4-6, upregulated in HPV-positive keratinocytes

Expression Cluster 4 (129 promoters)

matrix	# exp	freq exp	freq ran
ARNT_02	46	0.3566	0.2498
**CETS168_Q6**	44	0.3411	0.2485
**CETS1P54_03**	80	0.6202	0.4673
**E2F_Q3**	18	0.1395	0.0708
**E2F_Q3_01**	14	0.1085	0.0465
**E2F_Q4**	37	0.2868	0.1628
**E2F1_Q3_01**	20	0.1550	0.0877
**E2F1_Q6_01**	9	0.0698	0.0293
**ELK1_02**	65	0.5039	0.3871
ETF_Q6	67	0.5194	0.3982
GC_01	68	0.5271	0.3941
HSF2_01	10	0.0775	0.0260
KROX_Q6	34	0.2636	0.1800
**MEF2_02**	10	0.0775	0.0371
**MEF2_04**	20	0.1550	0.0762
MUSCLE_INI_B	53	0.4109	0.2933
MYC_Q2	38	0.2946	0.1837
SP1_Q2_01	47	0.3643	0.2687
SP1_Q4_01	56	0.4341	0.3186
SP1_Q6	51	0.3953	0.3007
USF_C	29	0.2248	0.1504
USF2_Q6	45	0.3488	0.2229
**VMYB_02**	79	0.6124	0.4720

Expression Cluster 5 (85 promoters)

matrix	# exp	freq exp	freq ran
**AP1FJ_Q2**	14	0.1647	0.0850
AP2_Q3	51	0.6000	0.4440
AP2_Q6	41	0.4824	0.3439
AP2_Q6_01	44	0.5176	0.3560
AP2ALPHA_01	22	0.2588	0.1618
**AR_02**	11	0.1294	0.0654
**AREB6_03**	17	0.2000	0.1035
ARNT_02	31	0.3647	0.2498
CP2_02	35	0.4118	0.2620
ETF_Q6	46	0.5412	0.3982
**ETS_Q4**	21	0.2471	0.1558
**GATA1_01**	32	0.3765	0.2633
GC_01	52	0.6118	0.3941
**GCM_Q2**	7	0.0824	0.0320
HSF1_Q6	1	0.0118	0.0003
LRF_Q2	36	0.4235	0.2471
**LUN1_01**	3	0.0353	0.0098
MAZ_Q6	42	0.4941	0.2980
**MEF3_B**	1	0.0118	0.0017
**MOVOB_01**	43	0.5059	0.3530
MYC_Q2	29	0.3412	0.1837
**MYCMAX_01**	6	0.0706	0.0243
MZF1_02	36	0.4235	0.2873
**NERF_Q2**	40	0.4706	0.3479
PAX5_01	53	0.6235	0.4673
POLY_C	1	0.0118	0.0003
**PR_01**	1	0.0118	0.0010
SP1_Q2_01	33	0.3882	0.2687
SP1_Q4_01	37	0.4353	0.3186
SP1_Q6	38	0.4471	0.3007
SP3_Q3	31	0.3647	0.2421
USF_C	25	0.2941	0.1504
**USF_Q6_01**	14	0.1647	0.0907
USF2_Q6	31	0.3647	0.2229

Expression Cluster 6 (8 promoters)

matrix	# exp	freq exp	freq ran
**CHX10_01**	1	0.125	0.0027
CP2_02	6	0.75	0.2620
**CREB_01**	5	0.625	0.2424
**CREB_Q2**	5	0.625	0.2205
**CREB_Q4**	5	0.625	0.2495
**CREB_Q4_01**	3	0.375	0.1150
**CREBP1_Q2**	5	0.625	0.2441
**CREBP1CJUN_01**	4	0.5	0.1625
**EVI1_06**	1	0.125	0.0105
**ICSBP_Q6**	2	0.25	0.0212
**IRF_Q6**	2	0.25	0.0169
**IRF2_01**	2	0.25	0.0270
**IRF7_01**	4	0.5	0.1015
**ISRE_01**	1	0.125	0.0078
**KAISO_01**	1	0.125	0.0172
LRF_Q2	5	0.625	0.2471
**MAF_Q6_01**	4	0.5	0.1962
**SRF_C**	1	0.125	0.0047
**SRF_Q5_01**	1	0.125	0.0088
**SRF_Q6**	1	0.125	0.0105

*TRANSFAC position weight matrices with a CORE_TF p-value for over-representation ≤ 0.01 and frequency in the random set < 50%.
Matrices unique to one expression cluster are in bold. Matrices shared between two expression clusters (either belonging to 1-3 or 4-6) are in bold and italics.
** Number of expression cluster promoters with a hit for the matrix.
*** Frequency of promoters with a hit for the matrix in 2966 random promoters
